# Quantitative electroencephalography as a potential biomarker in migraine

**DOI:** 10.1002/brb3.3282

**Published:** 2023-10-10

**Authors:** Suk Jae Kim, Kyungjin Yang, Daeyoung Kim

**Affiliations:** ^1^ Samsung Smart Neurology Clinic Cheonan Chungcheongnam‐do South Korea; ^2^ PE Research Lab, SK Hynix Inc. Icheon Gyeonggi‐do South Korea; ^3^ Department of Neurology Chungnam National University College of Medicine, Chungnam National University Hospital Daejeon South Korea

**Keywords:** diagnostic biomarker, migraine, quantitative electroencephalography, treatment monitoring

## Abstract

**Objective:**

The aim of this study was to investigate the utility of quantitative electroencephalography (QEEG) as a diagnostic tool for migraine and as an indicator of treatment response by comparing QEEG characteristics between migraine patients and controls, and monitoring changes in these characteristics alongside clinical symptoms in response to treatment

**Background:**

We hypothesized that patients with migraine exhibit distinctive characteristics in QEEG measurements, which could be used as potential diagnostic biomarkers and as a tool for monitoring treatment response.

**Methods:**

A total of 720 patients were included in the study, comprising 619 patients with migraine and 101 subjects as a control group. QEEG measurements were analyzed for absolute power across specific frequency bands: delta wave (0.5–4 Hz), theta wave (4–8 Hz), alpha wave (8–12 Hz), beta wave (12–25 Hz), and high beta wave (25–30 Hz). The absolute power was normalized against a normative dataset from NeuroGuide, with electrodes being highlighted for significance if they exceeded 1.96. Clinical symptoms were also monitored for correlation with QEEG changes.

**Results:**

Our analysis showed that patients with migraine exhibited significantly higher absolute power across all frequencies, most markedly within the high beta frequency range. When considering electrodes with *z*‐scores exceeding the threshold of 1.96 in the high beta range, a significant association with migraine diagnosis was observed (per 1 electrode increase, OR 1.06; 95% CI 1.01–1.11; *p* = .012). Moreover, pre‐ and posttreatment changes in QEEG measurements corresponded with changes in clinical symptoms.

**Conclusion:**

Patients with migraine have distinctive QEEG measurements, particularly regarding absolute power and the number of electrodes that surpassed the *z*‐score threshold in high beta wave activity. These findings suggest the potential of QEEG as a diagnostic biomarker and as a tool for monitoring treatment response in migraine patients, warranting further large‐scale studies for confirmation and expansion.

## Graphical abstract

Our study involved a large cohort of 619 patients with migraine, with a control group comprising both patients with occipital neuralgia and healthy subjects. We focused on QEEG measurements, specifically absolute power and the number of electrodes exceeding a certain threshold. Our results highlight significant differences in these measurements between the migraine and control groups. Furthermore, changes in these measurements correlated with changes in clinical symptoms, suggesting their potential use in monitoring treatment response.

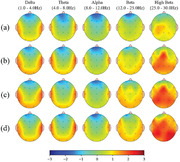

## 1 BACKGROUND

Migraine is a prevalent neurological disorder affecting a substantial proportion of the population (Ashina, Katsarava et al., 2021; Collaborators GBDH, 2018). However, the underlying mechanisms and pathophysiology of migraine are still poorly understood (Charles, 2018; Iyengar et al., 2019; Levy & Burstein, 2011; Wattiez et al., 2020). Recent research efforts have focused on identifying potential biomarkers for migraine, encompassing serologic, neurophysiologic, and neuroimaging markers (Ashina, Terwindt et al., 2021; Durham & Papapetropoulos, 2013; Schwedt et al., 2015). Despite these efforts, the identification and validation of clinically reliable and applicable biomarkers for migraine presents a significant challenge (Ashina, Terwindt et al., 2021).

In addition to the conventional electroencephalography (EEG) methods, various studies have utilized EEG event‐related potentials, somatosensory evoked potentials, and visual evoked potentials in the pursuit of potential biomarkers (Hsiao et al., 2023; 2021; Petrusic et al., 2021, 20222; Zhu et al., 2019). For instance, Zhu et al. (2019) demonstrated the efficacy of somatosensory evoked potentials for migraine classification. Petrusic et al. (2022) emphasized the relevance of P3 latency, particularly when understanding the complexity of migraine with aura. Furthermore, another study by Petrusic et al. (2021) presented findings on the N400 component in patients experiencing migraines with aura, hinting at the nuances of migraine pathophysiology.

Quantitative electroencephalography (QEEG) has been extensively utilized in the study of various neurological and psychiatric disorders (Geraedts et al., 2018; Kopanska et al., 2022; Walker, 2011). QEEG, which provides insights into brain function by analyzing and quantifying the electrical activity of the brain, serves as a valuable tool for clinical assessment and treatment (Newson & Thiagarajan, 2018). In the field of migraine research, QEEG has been employed in diverse studies, with objectives ranging from the exploration of pathophysiology to the monitoring of treatment efficacy (de Tommaso, 2019; Ojha & Panda, 2022; Walker, 2011).

In this study, our objective was to investigate the potential of QEEG as a biomarker for the diagnosis and monitoring of therapeutic effects in migraine patients. We hypothesized that patients with migraine exhibit distinct QEEG characteristics reflecting their unique pathophysiology, which may change in response to treatment.

## 2 MATERIALS AND METHODS

### 2.1 Subjects and grouping

We conducted a retrospective analysis using data from a prospective registry of patients who visited a headache clinic between March 2022 and February 2023. Participants were included in the migraine group if they had a diagnosis of migraine with or without aura based on the International Classification of Headache Disorders‐3 beta (ICHD‐3 beta) criteria, and in the disease control (DC) group if they had occipital neuralgia according to the ICHD‐3 beta (Headache Classification Committee of the International Headache Society (IHS), 2018). Individuals without any neurological or psychiatric disorders were included as the healthy control (HC) group. Subjects were excluded if they were younger than 18‐year old or did not undergo QEEG. The migraine group was further divided into three subtypes based on the headache frequency: low‐frequency episodic migraine (LF‐EM), defined as having fewer than 8 migraine headache days (MHD) per month; high‐frequency EM (HF‐EM), defined as having 8–14 MHD per month; chronic migraine (CM), defined as experiencing 15 or more headache days per month with at least 8 MHD per month (Headache Classification Committee of the International Headache Society (IHS), 2018; Jedynak et al., 2021). All patients provided written informed consent, and the local institutional review board approved this study.

### 2.2 Clinical evaluation, transcranial Doppler, and EEG recording

Patients were administered a structured headache questionnaire and underwent a clinical interview to assess age, sex, monthly headache days, and the use of analgesics and preventive medication. TCD and EEG studies were performed on the same day as the clinical evaluation by experienced neurophysiology technicians who were blinded to patient information. For the TCD study, the WAKI TCD (Atys Medical) was used. The mean flow velocities (MFVs) of the bilateral middle cerebral arteries (MCAs) were measured at four different depths (46–64 mm) and averaged to represent the MCA–MFVs. Our internal criteria defined MCA–MFV as increased if it exceeded 70 cm/s. The EEG recording was conducted in a laboratory that was specifically designed to minimize external noise and artifacts. The EEG signals of subjects were measured, whereas they were lying comfortably in bed but awake, using electrodes placed on the participant's scalp in accordance with the International 10/20 system. A reference electrode was attached to each ear lobe. The data were recorded for 5 min with eyes closed at a sampling rate of 256 Hz with Neuroworks (Natus Medical Inc.). A bandpass filter in the range of 0.5–70 Hz was applied to filter the EEG data, and a notch filter at 60 Hz was used to eliminate the artifact caused by the power supply during the EEG recording.

### 2.3 Quantitative EEG analysis

The EEG recordings from Neuroworks were transferred to NeuroGuide software, version 3.2.1.1 (Applied Neuroscience Inc.) for spectral analysis, using a linked ear montage. They were down‐sampled automatically to 128 Hz.

### 2.4 Artifact rejection and processing

To ensure the quality of the data, we implemented a rigorous artifact rejection process. The artifact rejection was applied to the continuous EEG data before segmenting it into the 2‐s epochs. This strategy was employed to ensure that our epochs remained artifact‐free, thus ensuring the accuracy of our subsequent analyses. First, we inspected the raw EEG traces visually and excluded the epochs with significant noncerebral activities, such as eye blinks, muscle activity, or electrical interference. Beyond manual inspection, we also utilized NeuroGuide's built‐in artifact rejection algorithms. These algorithms, designed for discerning well‐established artifact patterns like eye movements and blinks, combine time and frequency domain characteristics to sieve out non‐cerebral signals.

### 2.5 Spectral analysis and reliability assessment

Following artifact rejection, we applied the fast Fourier transform (FFT) to the artifact‐free continuous EEG data segmented into nonoverlapping 2‐second epoch. Only datasets where the sum of these segmented epochs equaled or exceeded 60 s were considered. This processing derived the absolute power from the frequency range of 0.5–30 Hz, using a frequency resolution of 0.5 Hz across 19 channels: FP1, FP2, F3, F4, F7, F8, C3, C4, T3, T4, T5, T6, P3, P4, O1, O2, Fz, Cz, and Pz. For reliability assessment, epochs with both a split‐half and a test–retest reliability greater than 0.90 for each EEG channel were included in the spectral analysis. The split‐half reliability correlated the power values of one half of the epochs with those of the other half. The test–retest reliability evaluated the consistency of power values analyzed twice within a single EEG recording session, ensuring consistent results from our analytical methods.

### 2.6 Computation of the *z*‐score and normative database

The absolute power was compared with a normative database from NeuroGuide. The *z*‐score was computed using the formula: (*z* = (*X* − *μ*)/*σ*), where *X* is the EEG power value of our subject, *μ* is the mean power value of the corresponding age group in the normative database, and *σ* is the standard deviation of the power values of the corresponding age group in the normative database. The normative database used from NeuroGuide consists of resting‐state EEG data collected from 625 individuals ranging in age from 2 months to 82.6 years with eyes closed, mirroring the conditions of our study. The EEG channels in the normative dataset align with the 19 channels used in our research. Both our study and the NeuroGuide database use referencing to linked earlobes. This methodological consistency ensures that our normalization based on the normative data is valid and directly comparable.

### 2.7 Frequency band analysis and *z*‐score thresholding

The EEG data were categorized into specific frequency bands representing distinct brain activities: delta wave (0.5–4 Hz), theta wave (4–8 Hz), alpha wave (8–12 Hz), beta wave (12–25 Hz), and high beta wave (25–30 Hz). For an individual subject, the *z*‐scores from the 19 electrodes were averaged to represent the mean *z*‐score of each wave. To determine individual electrode activity in each band, electrodes were considered to exceed the threshold if their *z*‐score of FFT absolute power exceeded 1.96, which corresponds to the top 2.5% of the normative database from NeuroGuide.

### 2.8 Follow‐up

In the migraine group, preventative medications—such as valproic acid, topiramate, flunarizine, amitriptyline, or propranolol—were prescribed as necessary. Follow‐up QEEGs were performed on certain patients whose clinical conditions either improved or worsened, particularly when there was a change of 50% or more in the frequency or intensity of headaches. These follow‐up QEEG results were then compared to the baseline QEEGs.

### 2.9 Statistical analysis

Statistical analyses were performed using the commercially available software package, SPSS Statistics version 27.0 (IBM Corp.). All data are presented either as the median (25th–75th percentile) or as a number (percentage). The Shapiro–Wilk test was employed to test the normal distribution of continuous variables. Given that the distributions were found to be non‐normal (*p* < .05), we used the Mann–Whitney *U*‐test to compare continuous variables between groups. Pearson's chi‐square or Fisher's exact test was utilized for the comparison of categorical variables. To compare the initial and follow‐up QEEG findings, we employed the Wilcoxon signed‐rank test.

A multivariable logistic regression analysis was performed to predict the independent contribution of factors in the migraine versus DC group. Potential explanatory variables, including age, sex, increased MCA–MFV on TCD, and the number of electrodes exceeding the threshold in high beta wave in QEEG, were simultaneously entered into multivariable models. We considered a *p*‐value of <.05 to be statistically significant.

## 3 RESULTS

**FIGURE 1:**
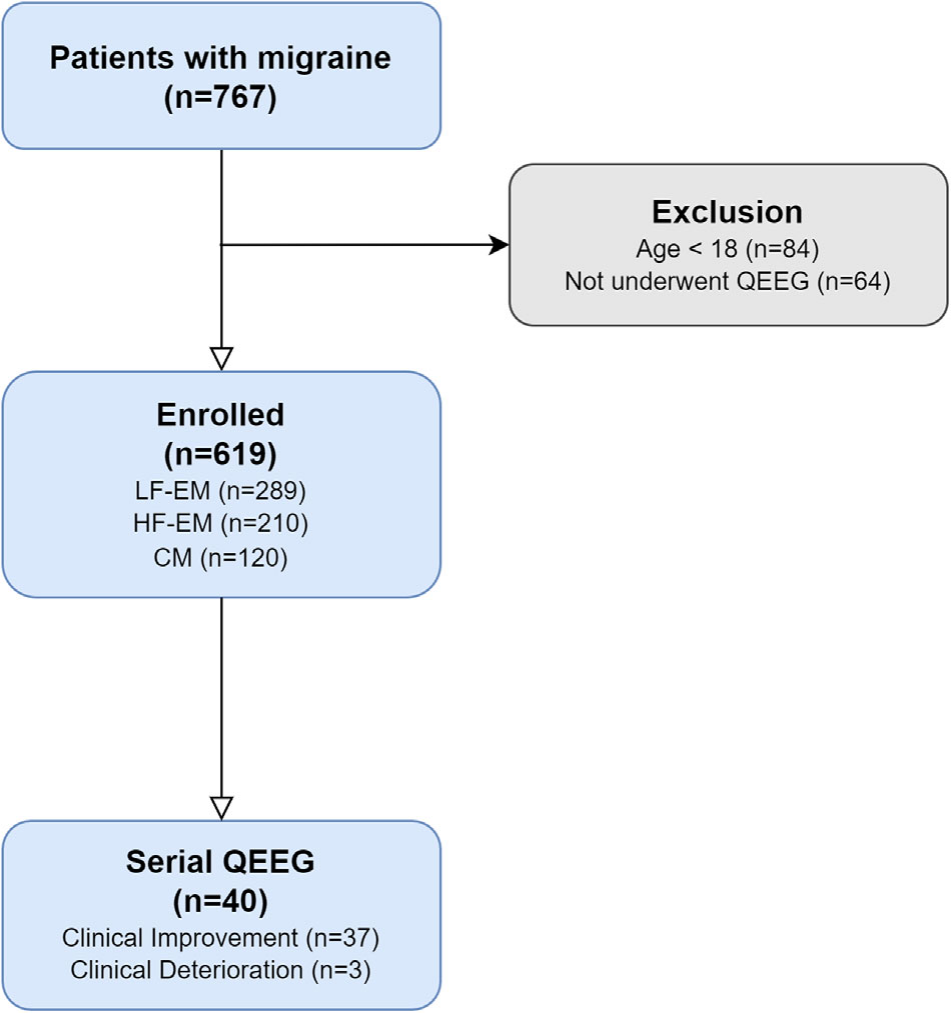
Overall study flow. CM, chronic migraine; HF‐EM, high‐frequency episodic migraine; LF‐EM, low‐frequency episodic migraine; QEEG, quantitative electroencephalography.

During the study period, we enrolled a total of 619 patients with migraines, including 191 men and 428 women. Of these, 289 (46.7%) had LF‐EM, 210 (33.9%) had HF‐EM, and 120 (19.4%) had CM. The median patient age was 37 years (IQR 30–46). We also included 86 patients from the DC group and 15 individuals from the HC group as controls in the study. Figure 1 depicts the overall flow of the study.

Table 1 presents the baseline characteristics of the migraine and control groups. Among the migraine patients included in the study, the majority were characterized by the absence of aura, had not undergone prior treatment with migraine prophylactic medication, and had their EEG recordings taken during the interictal phase. The migraine group had a significantly higher proportion of female patients (*p* < .001) and a younger age distribution (*p* = .027) compared to the control group. We performed TCD on 560 (90.5%) patients in the migraine group and 74 (86.0%) patients in the DC group. The TCD findings demonstrated significantly higher median MCA–MFVs in the migraine group compared to the DC group (*p* = .005). However, the difference between the two groups became nonsignificant when compared based on the proportion of patients with increased MCA–MFVs (*p* = .379).

**TABLE 1:** Baseline characteristics.

	Migraine group (*n* = 619)				Control group (*n* = 101)			*p*‐Value
	LF‐EM (*n* = 289)	HF‐EM (*n* = 210)	CM (*n* = 120)	Total	DC (*n* = 86)	HC (*n* = 15)	Total	Migraine vs. control
Age, years (IQR)	36 (31–45)	37 (29–46)	41 (33–51)	37 (30–46)	40 (35–50)	34 (25–43)	40 (34–49)	.027
Female, *n* (%)	188 (65.1)	152 (72.4)	88 (73.3)	428 (69.1)	42 (48.8)	8 (53.3)	50 (49.5)	<.001
Migraine characteristics								
**Migraine attack during** **EEG recording, *n* (%)**	24 (8.3)	22 (10.5)	13 (10.8)	59 (9.5)	NA	NA	NA	NA
**Migraine without aura, *n* (%)**	259 (89.6)	186 (88.6)	106 (88.3)	551 (89.0)	NA	NA	NA	NA
**Naïve to migraine prophylactic medication, *n* (%)**	289 (100)	202 (96.2)	109 (90.8)	600 (96.9)	NA	NA	NA	NA
TCD findings (*n* = 634)^a^	*n* **= 267**	*n* **= 187**	*n* **= 106**	*n* **= 560**	*n* **= 74**		*n* **= 74**	
**MCA MFV (cm/s)**	70 (63–80)	70 (62–80)	69 (60–79)	70 (62–80)	65 (56–74)	NA	65 (56–74)	.005
**Increased MFV, *n* (%)**	123 (46.1)	80 (42.8)	39 (36.8)	242 (43.2)	28 (37.8)	NA	28 (37.8)	.379
QEEG findings								
Mean *z*‐score								
**Delta**	0.56 (0.24–1.36)	0.62 (0.14–1.32)	0.60 (0.04–1.13)	0.59 (0.23–1.29)	0.35 (−0.14–1.01)	0.46 (−0.27–0.83)	0.36 (−0.14–0.96)	.019
**Theta**	0.50 (−0.16–1.15)	0.44 (−0.10–1.13)	0.53 (0.05–1.09)	0.48 (−0.10–1.13)	0.11 (−0.26–0.66)	0.00 (−0.39–0.79)	0.10 (−0.30–0.67)	<.001
**Alpha**	0.42 (−0.38–0.59)	0.12 (−0.36–0.71)	0.18 (−0.42–0.68)	0.10 (−0.38–0.64)	−0.08 (−0.49–0.36)	−0.37 (−0.99–0.58)	−0.09 (−0.54–0.34)	.008
**Beta**	0.68 (−0.04–1.60)	0.82 (0.34–1.67)	0.87 (0.14–1.82)	0.77 (0.13–1.67)	0.38 (−0.26–0.88)	0.23 (−0.39–0.59)	0.34 (−0.27–0.86)	<.001
**High beta**	1.44 (0.59–3.26)	1.73 (0.82–3.22)	1.72 (0.68–3.31)	1.57 (0.65–3.25)	0.65 (0.14–1.84)	0.81 (0.10–1.63)	0.76 (0.13–1.77)	<.001
Number of electrodes^b^								
**Delta**	1 (0–5)	2 (0–5)	1 (0–4)	1 (0–5)	1 (0–3)	1 (0–2)	1 (0–2)	.006
**Theta**	1 (0–5)	1 (0–4)	1 (0–4)	1 (0–4)	0 (0–2)	0 (0–2)	0 (0–2)	<.001
**Alpha**	0 (0–1)	0 (0–2)	0 (0–1)	0 (0–1)	0 (0–1)	0 (0–1)	0 (0–1)	.059
**Beta**	2 (0–6)	2 (0–7)	2 (0–7)	2 (0–7)	0 (0–2)	0 (0–2)	0 (0–2)	<.001
**High beta**	5 (1–13)	6 (2–13)	8 (2–15)	6 (2–13)	3 (0–6)	3 (0–4)	3 (0–6)	<.001

Abbreviation: CM, chronic migraine; DC, disease control; EEG, electroencephalography; HF‐EM, high‐frequency episodic migraine; HC, healthy control; IQR, interquartile range; LF‐EM, low‐frequency episodic migraine; MCA, middle cerebral artery; MFV: mean flow velocity; QEEG, quantitative electroencephalography; TCD, transcranial Doppler.

^a^ A total of 59 patients (9.5%) in the migraine group and 12 patients (14.0%) in the DC group were unable to undergo TCD examination mainly due to poor temporal windows.

^b^ The number of electrodes where the QEEG signal had a *z*‐score greater than 1.96.

**FIGURE 2:**
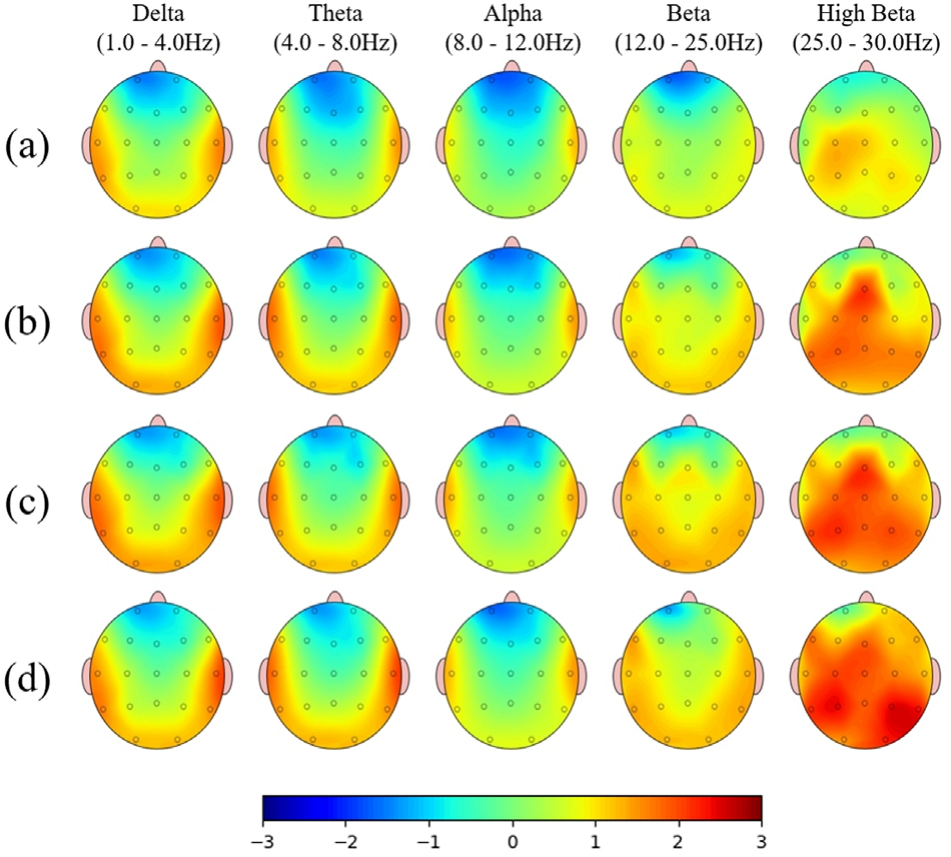
Representative quantitative electroencephalography (QEEG) patterns across subgroups, based on the median value of *z*‐scores measured at each electrode across subjects. The depicted QEEG images show the 19 electrodes affixed to the scalp, each colored according to the corresponding *z*‐score. The color scale ranges from blue (indicating lower *z*‐scores) to light green (*z*‐scores approaching zero) and then to red for higher *z*‐scores. (a) Control, (b) LF‐EM (low‐frequency episodic migraine), (c) HF‐EM (high‐frequency episodic migraine), and (d) CM (chronic migraine).

In terms of QEEG findings, we compared the mean *z*‐scores of delta, theta, alpha, beta, and high beta absolute power across all electrodes in the brain for each individual participant. The results showed that in all frequency bands, the migraine group had significantly higher *z*‐scores compared to the control group (*p* = .019 for delta wave, *p* < .001 for theta wave, *p* = .008 for alpha wave, *p* < .001 for beta wave, and *p* < .001 for high beta wave, respectively). Similarly, we compared the number of electrodes exceeding the threshold in each frequency range between the two groups. Except for the alpha wave, the migraine group had a significantly higher number of electrodes exceeding the threshold compared to the control group in all frequency ranges (*p* = .006 for delta wave, *p* < .001 for theta wave, *p* = .059 for alpha wave, *p* < .001 for beta wave, and *p* < .001 for high beta wave, respectively). In particular, in the high beta frequency range, the migraine group showed a tendency of increasing median values of the number of electrodes exceeding the threshold in the order of LF‐EM, HF‐EM, and CM. There was no difference in the median values of this parameter between the DC and HC in the control group. Figure 2 illustrates the representative QEEG patterns for each subgroup, based on the median value of *z*‐scores measured at each electrode across subjects.

Table 2 demonstrates the results of the multivariate logistic regression analysis conducted to evaluate independent predictors for the migraine versus DC group. Being female (OR 2.32; 95% CI 1.37–3.94; *p* = .002) and the number of electrodes exceeding the threshold in high beta wave in QEEG (per 1 electrode increase, OR 1.06; 95% CI 1.01–1.11; *p* = .012) were independently associated with migraine. Other factors, including age and increased MCA–MFVs, did not contribute significantly to predicting migraine.

**TABLE 2:** Multiple logistic regression analysis: independent predictors for migraine versus control.

	Crude OR	Multivariate testing	
		OR (95% CI)	*p*‐Value
Age, per 1 year increase	0.98 (0.97–1.00)	0.98 (0.96–1.00)	.051
Sex	2.29 (1.49–3.50)	2.32 (1.37–3.94)	.002
Increased MFV on TCD	1.25 (0.76–2.06)	0.85 (0.49–1.48)	.566
Number of electrodes of high beta on QEEG^a^, per 1 electrode increase	1.09 (1.05–1.13)	1.06 (1.01–1.11)	.012

Abbreviation: CI, confidence interval; MFV: mean flow velocity; OR, odds ratio; QEEG, quantitative electroencephalography; TCD, transcranial Doppler.

^a^ The number of electrodes where the QEEG signal had a *z*‐score greater than 1.96.

Among the patients with migraine, 40 underwent follow‐up QEEG to monitor the response to treatment. Of these, 37 demonstrated clinical improvement (6 LF‐EM, 17 HF‐EM, and 14 CM), whereas 3 exhibited clinical deterioration (1 LF‐EM, 1 HF‐EM, and 1 CM). The median interval between QEEG tests was 56 days (IQR 36–110 days). Table 3 reveals that the mean *z*‐scores of delta, alpha, beta, and high beta absolute power significantly decreased as clinical improvement was observed (*p* = .009 for delta wave, *p* = .023 for alpha wave, *p* = .026 for beta wave, and *p* = .024 for high beta wave, respectively). In a similar manner, the number of electrodes surpassing the threshold in the delta, beta, and high beta ranges significantly decreased in patients with clinical improvement, with the most prominent reduction observed in the high beta range (*p* = .032 for delta wave, *p* = .016 for beta wave, and *p* = .001 for high beta wave, respectively).

**TABLE 3:** Changes in mean *z*‐scores and number of electrodes exceeding the threshold in different EEG frequency bands associated with clinical improvement.

	Pretreatment	Posttreatment	Difference	*p*‐Value
Mean *z*‐score on QEEG				
Delta	0.95 (0.59–1.61)	0.39 (−0.18–1.27)	0.55 (−0.23–1.28)	.009
Theta	0.84 (0.28–1.56)	0.52 (−0.25–1.03)	0.33 (−0.20–0.78)	.060
Alpha	0.32 (−0.10–1.06)	0.02 (−0.55–0.81)	0.25 (−0.13–0.68)	.023
Beta	1.45 (1.02–2.33)	0.76 (0.30–1.68)	0.45 (−0.32–1.26)	.026
High beta	2.74 (1.59–4.32)	1.37 (0.86–2.93)	0.91 (−0.45–2.51)	.024
Number of electrodes on QEEG^a^				
Delta	4 (1–9)	1 (0–6)	1 (−0.5–3.5)	.032
Theta	3 (0.5–8)	1 (0–6)	0 (−1–3)	.354
Alpha	1 (0–3)	0 (0–2)	0 (0–1)	.877
Beta	6 (2–13)	2 (1–6.5)	1 (−1–7.5)	.016
High beta	13 (8–17)	6 (3–13)	3 (−0.5–11)	.001

Abbreviation: EEG, electroencephalography; QEEG, quantitative electroencephalography.

^a^ The number of electrodes where the QEEG signal had a *z*‐score greater than 1.96.

**FIGURE 3:**
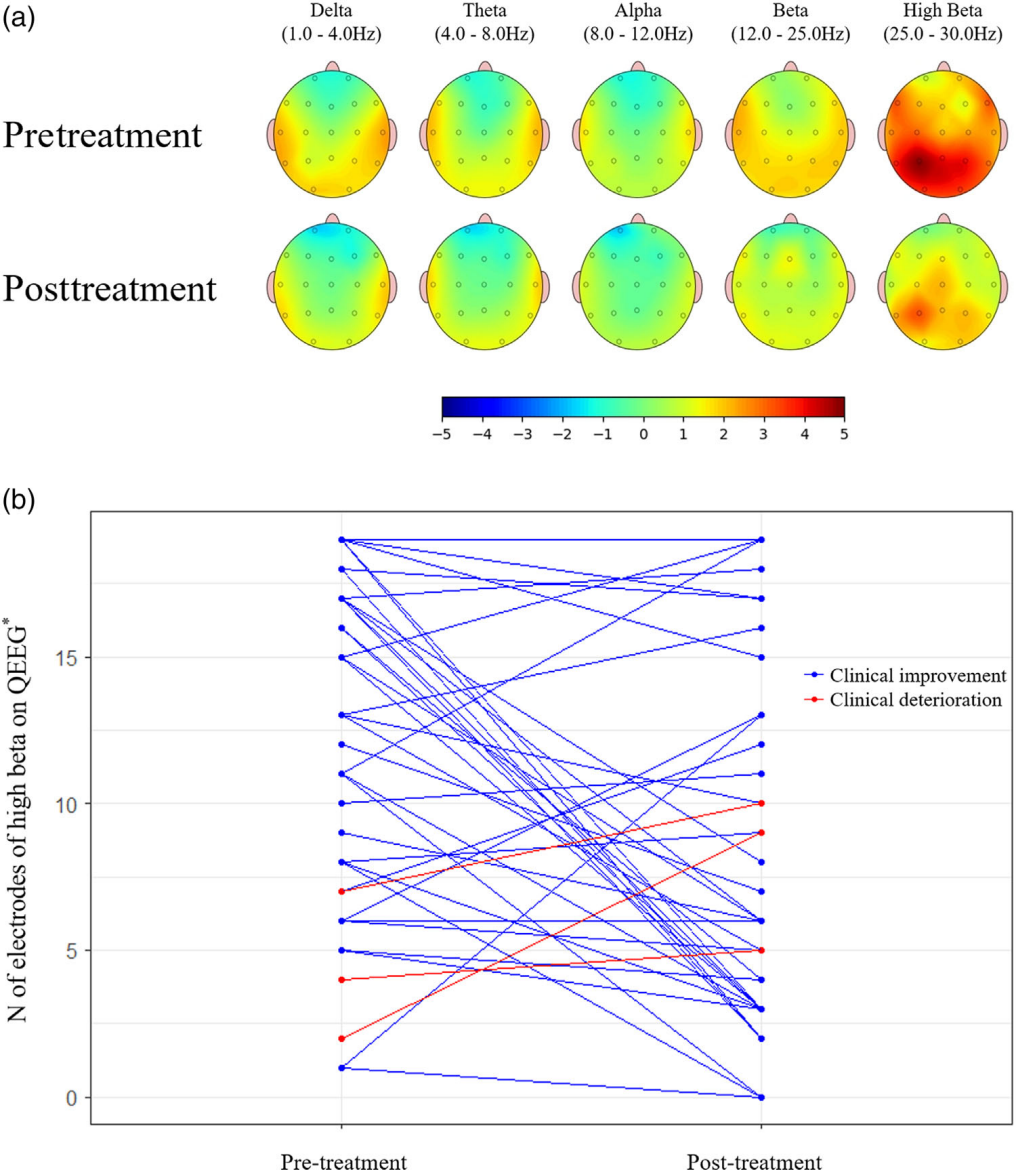
Serial quantitative electroencephalographic (QEEG) alterations (a) QEEG modifications before and after treatment, based on the median value of *z*‐scores measured at each electrode across subjects showing clinical improvement. (b) Differential shifts in the number of high beta frequency band electrodes surpassing the predetermined threshold among patients demonstrating clinical improvement versus deterioration. *Note*: The number of electrodes where the QEEG signal had a *z*‐score greater than 1.96.

Figure 3a provides a graphical depiction of the changes in serial qEEG. Figure 3b, on the other hand, shows the fluctuations in the number of electrodes exceeding the predetermined threshold in the high beta frequency band for patients who showed clinical improvement versus those who demonstrated clinical deterioration. Of the patients who showed clinical improvement, 70.3% (26 out of 37) demonstrated a decrease in the number of electrodes surpassing the threshold. On the contrary, all patients demonstrating clinical deterioration showed an increase in the number of electrodes exceeding the threshold.

## 4 DISCUSSION

The major findings of the current study are (a) patients with migraine showed a tendency for an increase in absolute power in all frequencies on QEEG compared to the control group. In particular, the number of electrodes that exceeded the threshold in high beta wave was independently associated with migraine and (b) the observed alterations in QEEG between the pre‐ and posttreatment measurements appeared to correspond to changes in clinical symptoms.

Migraine is primarily diagnosed through clinical history, and the utilization of neuroimaging techniques, such as computed tomography and magnetic resonance imaging, is intended to exclude secondary headaches (Marmura et al., 2015). Neurophysiological tests, including QEEG and TCD, are being explored as potential diagnostic biomarkers for migraine, reflecting its pathophysiology (Arjona et al., 2007; de Tommaso, 2019). In this study, sex and QEEG findings were found to have independent associations with migraine compared to the control group. It is well known and consistent with previous research that there are more women with migraine compared to occipital neuralgia, which was selected as the control group (Ashina, Katsarava et al., 2021; Koopman et al., 2009).

There is an association between migraine attacks and increased brain hyperexcitability, with attacks more likely to occur when the brain is hypersensitive, and prolonged attacks more likely to increase neuronal hyperexcitability (Aurora & Wilkinson, 2007; Charles, 2018; Noseda & Burstein, 2013). QEEG can measure neuronal hyperexcitability by analyzing the frequency, amplitude, and phase characteristics of EEG signals using various mathematical and statistical techniques to assess brain activity (de Tommaso, 2019). Of the parameters available, spectral analysis of absolute power has been emphasized in previous research as a sensitive index for identifying cortical abnormalities (Liu et al., 2021; Minnerly et al., 2021). By quantifying the total electrical activity generated by the brain, absolute power provides valuable information when investigating conditions like migraines that are potentially linked to altered neural excitability (Bjork & Sand, 2008; Walker, 2011). Given its simplicity and interpretability compared to more complex parameters, such as spectral entropy or cross‐frequency coupling, absolute power measurements are particularly advantageous in clinical settings where clear communication of findings with patients and practitioners is essential (Zhang et al., 2023).

The present study aimed to address some of the limitations of using absolute power in EEG studies. Although previous studies predominantly analyzed raw data of absolute power and compared it across individual electrodes, this approach can be complex and impractical for routine clinical use (Bjork et al., 2009; Kwan et al., 2018). Hence, we employed a strategy of using *z*‐scores of absolute power values, which enabled us to effectively adjust for age that may influence absolute power (Thatcher, 1998). Moreover, with an eye toward enhancing the practicality and interpretability of our findings, we counted the number of electrodes that exceeded the threshold, rather than solely focusing on individual electrode power. This approach offers a more straightforward way to visualize and understand the extent of deviation from the norm across the entire brain region being monitored. It also ensures that our findings are not unduly influenced by outliers in the data or disproportionate contributions from individual electrodes. This pragmatic and inclusive method enhances the usability of our findings in a clinical setting, providing a clear and accessible indicator of aberrant EEG activity.

The significant elevation of absolute power across all frequencies in migraine patients relative to control subjects could be indicative of a state of generalized cortical hyperactivity or hypersensitivity that is characteristic of migraines (Gollion, 2021; Gomez‐Pilar et al., 2020). However, previous research in this area has yielded a spectrum of findings, with some results resonating with ours and others presenting divergent patterns (Zhang et al., 2023). This diversity should be understood in light of the varied methodologies employed across these studies. Specific methodological elements, such as the selection of frequency bands examined, the use of normalization techniques like the conversion of absolute power values into *z*‐scores, the demographic and clinical characteristics of the patient populations studied, and the attributes of the control cohorts, can significantly impact the findings (de Tommaso, 2019).

In this study, the specific selection of high beta frequency data for regression analysis was motivated by two key factors. First, there exists substantial literature suggesting a strong link between increased power in higher frequency bands, such as the high beta range, and heightened neuronal hypersensitivity (Das & Yadav, 2020; Kwan et al., 2018; Walker, 2011). Moreover, within our own dataset, the difference in power between the migraine and control groups was most marked within the high beta frequency range. Notably, we observed a progressive relationship, where the degree of power elevation increased systematically from LF‐EM, to HF‐EM, and finally to CM. This pattern may indicate a correlation between the level of neuronal hyperexcitability and the severity or frequency of migraine attacks, providing further justification for our emphasis on the high beta frequency in the regression analysis (Gomez‐Pilar et al., 2020).

Although our study on the utilization of QEEG for monitoring treatment response is promising, it is important to interpret these findings with caution due to the small sample size and potential selection bias. Nonetheless, the observed reductions in the mean *z*‐scores of delta, alpha, beta, and high beta absolute power in conjunction with clinical improvements provide preliminary evidence of a potential correlation between QEEG parameters and patient outcomes. The observed decrease in the number of electrodes exceeding the threshold, particularly within the high beta range, for patients demonstrating clinical improvement, juxtaposed with an increase in the same parameter for those with clinical deterioration, tentatively suggests QEEG could serve as a sensitive biomarker for monitoring treatment progress (Sneddon et al., 2006). Additionally, one of the prominent challenges associated with QEEG absolute power analysis—the potential impact of thickness and properties of the skull and other tissues of the head—becomes negligible when comparing pre‐ and posttreatment data within the same individual.

Controversies regarding the role of TCD in migraine notwithstanding, some previous studies have shown moderately higher basal cerebral artery MFVs in migraine patients compared to controls, consistent with our findings (Arjona et al., 2007; Dzator et al., 2021; Khedr et al., 2022). However, the relatively small difference in MCA–MFVs between the migraine and control groups, despite achieving statistical significance, implies that TCD may not be a highly effective diagnostic biomarker (Kastrup et al., 1998; Thie et al., 1990). Furthermore, the absence of a significant difference in the number of patients with elevated MCA–MFVs limits the usefulness of TCD as a diagnostic biomarker (DeCoster et al., 2011).

The study possesses certain strengths that underscore the value of its findings. In particular, the inclusion of a relatively large patient cohort, consecutively enrolled, and the consistent clinical work‐up performed on most patients add substantial credibility. Further validity is lent by the selection of a control group with occipital neuralgia, a condition of peripheral rather than central origin, which provided insightful comparative data. Confirming the appropriateness of our control selection, we found no significant difference in QEEG findings between genuinely healthy individuals and those with occipital neuralgia.

Despite these promising findings, it is essential to acknowledge the limitations of our study. Given its single‐center design, our findings might lack broader applicability, suggesting the need for multicenter studies for more robust and generalizable conclusions. Furthermore, potential confounding variables, such as coexisting medical conditions, use of medications, and the state of migraine (whether ictal or interictal) at the time of QEEG recording, were not controlled in our analysis. These factors can influence QEEG results and therefore should be considered and adjusted for in future research, to enhance the understanding and clinical utility of QEEG in diagnosing and managing migraine.

In conclusion, our findings suggest a potential use of QEEG in differentiating migraine patients from controls and in monitoring treatment response. However, additional, larger scale studies considering potential confounding factors are necessary to corroborate and expand upon these preliminary insights.

## AUTHOR CONTRIBUTION

**Suk Jae Kim**: Conceptualization; data acquisition; analyzed and interpretation of data; writing—original draft; writing—review and editing. **Kyungjin Yang**: Conceptualization; data acquisition; analyzed and interpretation of data; writing—review and editing. **Daeyoung Kim**: Conceptualization; analyzed and interpretation of data; writing—review and editing.

## FUNDING INFORMATION

No funding was received for this study.

### PEER REVIEW

The peer review history for this article is available at https://publons.com/publon/10.1002/brb3.3282.

## Data Availability

The data that support the findings of this study are available from the corresponding author upon reasonable request.
